# Therapeutic Outcomes of Patients with Multifocal Papillary Thyroid Microcarcinomas and Larger Tumors

**DOI:** 10.1155/2017/4208178

**Published:** 2017-05-31

**Authors:** Soh-Ching Ng, Sheng-Fong Kuo, Szu-Tah Chen, Chuen Hsueh, Bie-Yu Huang, Jen-Der Lin

**Affiliations:** ^1^Division of Endocrinology and Metabolism, Department of Internal Medicine, Chang Gung Memorial Hospital, Keelung, Taiwan; ^2^Division of Endocrinology and Metabolism, Department of Internal Medicine, Chang Gung Memorial Hospital and Chang Gung University, Keelung, Taiwan; ^3^Department of Pathology, Chang Gung Memorial Hospital and Chang Gung University, Keelung, Taiwan

## Abstract

A retrospective review of 626 patients with multifocal papillary thyroid carcinoma (PTC) including 147 patients (23.5%) with multifocal papillary thyroid microcarcinoma (PTMC) from a total of 2,536 patients with PTC who visited the Chang Gung Medical Center in Linkou, Taiwan, was performed. A comparison of the clinical features between 626 multifocal and 1,910 solitary PTC cases showed that patients in the multifocal PTC group were older and had a smaller mean tumor size, a more advanced tumor-node-metastasis (TNM) stage, and a higher percentage of nonremission status compared to patients in the solitary PTC group. Of the 626 patients with multifocal PTC, the group with larger tumors showed a more advanced TNM stage, a higher percentage of lymph node metastasis and soft tissue invasion, and a higher nonremission rate compared to the multifocal PTMC group. Of the 626 patients with multifocal PTC, 25 patients (4%) died during a mean follow-up period of 7.1 ± 5.3 years. Kaplan-Meier survival curves showed a significantly lower survival rate associated with multifocal PTMC compared to that with solitary PTMC.

## 1. Introduction

Multifocal papillary thyroid carcinoma (PTC) may present as microcarcinoma (≤1.0 cm) or larger tumors in two or more individual locations within the thyroid gland. An increase in the incidence of papillary thyroid microcarcinoma (PTMC) and multifocal PTC has been reported in the recent decade [1–3]. Intrathyroid multifocal PTMC is categorized as a low-risk group in recent American Thyroid Association (ATA) guidelines [4]. In contrast, multifocal PTMC with extrathyroidal extensions is categorized as an intermediate-risk group. Bilateral multifocal PTMC has a worse prognosis than unilateral PTMC [5, 6]. Multifocal PTC may be diagnosed as “incidental” in the final histopathology examination or “nonincidental” if it is diagnosed before or during thyroid surgery [7, 8]. For both incidental and nonincidental PTCs, prognostic factors of these patients are important to determine whether a second surgery for complete total thyroidectomy is necessary. Postoperative radioiodine (^131^I) remnant ablation and other imaging techniques are important for identifying patients with a high risk of incidental or nonincidental PTC [9]. However, data are lacking concerning the long-term therapeutic outcomes of patients with multifocal PTMC and multifocal PTC with larger tumors in order to provide appropriate therapeutic modalities for these patient groups. Analyses of long-term follow-up results after multiple modality treatments are important and may provide better therapeutic strategies for the treatment of patients with multifocal PTC.

The aims of this study were to investigate the long-term follow-up outcomes of patients after different therapeutic strategies for the treatment of multifocal PTC including PTMC and larger tumors. We analyzed different therapeutic strategies including surgical methods, ^131^I remnant ablation, ^131^I treatment, imaging studies, and external radiotherapy. Various therapeutic strategies and risk factors for cancer mortality and recurrence in different patient groups were analyzed.

## 2. Materials and Methods

**Figure 1:**
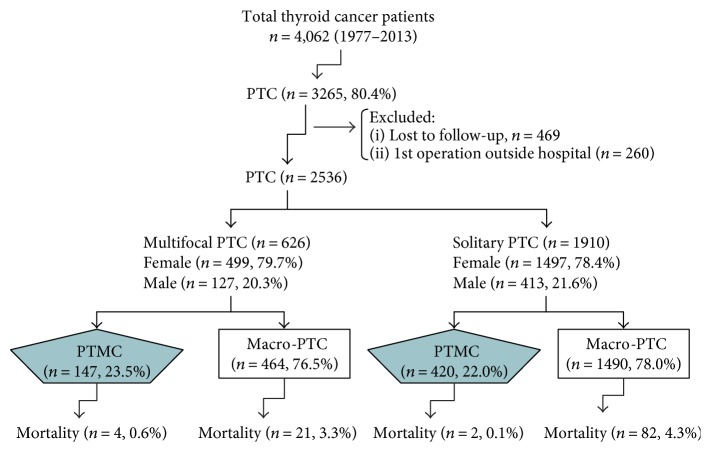
Distribution of papillary thyroid carcinoma patients in the current study. PTC: papillary thyroid cancer; PTMC: papillary thyroid microcarcinoma.

We selected 3265 patients with PTC from a total of 4062 patients with thyroid cancer who had undergone thyroid surgery between 1977 and 2013 at the Chang Gung Medical Center in Linkou, Taiwan. Patients who did not undergo a follow-up for over 1 year and who underwent the initial thyroid surgery at a different hospital and patients without data regarding tumor sizes were excluded from our analyses (Figure 1). All patients had undergone primary thyroid surgery and a long-term clinical follow-up at Chang Gung Medical Center. Frozen tissue specimens from 525 patients were obtained during surgery and evaluated by pathologists. Initially, each specimen was assessed macroscopically to identify the most significant nodules and the most invasive tumors and to plan the surgical dissection of the surgical sample. Intraoperative examinations of the frozen section were performed on the cut surface of the thyroid nodules including the interface between the nodule and adjacent thyroid tissue. A total thyroidectomy was performed on patients with PTC with extrathyroidal extensions and lymph node metastasis. Patients with PTMC and an absence of extrathyroidal invasion as diagnosed by postoperative histopathology underwent follow-up care if they had undergone a subtotal thyroidectomy. Subtotal thyroidectomy was defined as removing more than 50% of the entire gland on both lobes. One hundred and forty patients received secondary thyroid operations for a complete thyroidectomy after PTC was confirmed by histology.

A total of 2536 PTC patients were enrolled in this study, including 626 patients with multifocal PTC and 147 patients with multifocal PTMC. In contrast, there were 1910 patients with solitary PTC including 420 (22.0%) patients with papillary microcarcinomas. After thyroid surgery, patients were staged using the Union for International Cancer Control tumor-node-metastasis (TNM) criteria (6th edition) [10]. All thyroid carcinoma tissues were pathologically classified according to the World Health Organization criteria [11]. PTMC was defined as the largest tumor diameter ≤ 1 cm in the final histological slides. Cases were defined as multifocal if two or more isolated foci of PTC were found.

In our center, patients with PTC at high or intermediate risk were recommended to undergo thyroid ^131^I remnant ablation 4 to 6 weeks after thyroidectomy [4]. The dose of ^131^I ablation for most patients was 30–100 mCi (1.1–3.7 GBq). One week after ^131^I administration, a whole-body scan (WBS) was performed using a dual-head gamma camera (Siemens Medical Solutions USA Inc., USA). Thyroid scintigraphy was performed using a pinhole collimator with a 4 mm aperture placed 7 cm above the neck for a total of 50,000 counts for 30 minutes. Levothyroxine treatment was initiated to decrease the levels of thyroid-stimulating hormone without inducing clinical thyrotoxicosis. If ^131^I uptake extended beyond the thyroid bed, patients were classified as having residual disease or metastasis unless proven to be a false-positive result. Higher therapeutic doses of 3.7–7.4 GBq (100–200 mCi) were administered to these patients. Patients receiving doses exceeding 1.1 GBq were isolated at hospital admission. A WBS was performed 2 weeks after administering higher therapeutic doses of ^131^I. Neck ultrasonography was performed 6–12 months after thyroidectomy to exclude the possibility of local recurrence.

At the end of 2014, patients were categorized into four groups: thyroid cancer mortality, nonremission, remission, and disease-free. The remission group consisted of patients with negative ^131^I WBS results and no evidence of local or distant metastasis upon noninvasive examination. Disease-free patients were defined as patients in remission with undetectable levels of stimulated thyroglobulin (Tg) and undetectable Tg antibodies at the final follow-up appointment.

The Chang Gung Medical Foundation Institutional Review Board approved this study (104-3901B); the requirement for informed consent was waived because of the retrospective nature of the study.

In our hospital, serum Tg levels were measured using an immunoradiometric assay kit (CIS Bio International, Gif-sur-Yvette, France) before the end of 2014. The detection limit of the Tg kit was 0.5 ng/mL. The functional sensitivity of this assay, as assessed in our laboratory, was 1.2 ng/mL. Tg antibody levels were measured using a competitive radioimmunoassay (Biocode, Liège, Belgium) with an analytical sensitivity of 6 IU/mL.

Unpaired *t*-tests were used to compare continuous data between groups. Categorical data were compared using chi-square or Fisher's exact tests for small data sets. Cancer-related mortality was calculated, and the follow-up period was determined from the date of diagnosis to the date of cancer-related mortality of the last survivor undergoing follow-up care. Survival rates were calculated using the Kaplan-Meier method and compared using a log-rank test [12]. A multivariate Cox proportional hazard regression model was used to estimate the mortality risk. All statistical analyses were performed using the SPSS software, version 17.0 (SPSS Inc., Chicago, IL, USA). *p* values < 0.05 were considered statistically significant in all tests.

## 3. Results

Table 1 illustrates the clinical features of the 2536 patients with PTC categorized into a multifocal group (626 patients, 24.7%) and a solitary tumor (1910 patients, 75.3%) group. Patients in the multifocal PTC group were older, had a smaller mean tumor size and a higher percentage, had undergone a total or complete thyroidectomy, and showed more advanced clinical and TNM stages; a higher percentage of patients were in nonremission. In addition, the multifocal PTC group had a higher mean postoperative accumulated ^131^I dose and a lower percentage in disease-free status compared to the solitary PTC group. In contrast, sex, postoperative serum Tg levels, histological variants of PTC, thyroid cancer-specific mortality, and total mortality did not differ significantly between these two groups.

Of the 626 patients with multifocal PTC, 147 (23.5%) patients had tumors of less than or equal to 1.0 cm. The group with larger tumors had a significantly higher percentage of patients that had undergone a total thyroidectomy, had a more advanced TNM stage, had a higher percentage of lymph node metastasis and soft tissue invasion, and had a higher nonremission rate than the patient group with multifocal PTMC (Table 2). Age, sex, disease-specific mortality, overall mortality, and disease-free status did not differ significantly between these groups. The follicular variant of PTC was observed in 15.8% of all multifocal PTC diagnoses. The larger tumor group had a higher percentage of follicular variant PTC compared to the microcarcinoma group (17.7% versus 9.5%; *p* = 0.017).

Of the 626 patients, 31 (5.0%) underwent subtotal or lobectomy. Most of these patients presented with small tumor sizes and a less advanced TNM stage. There were single patients with lymph node metastasis, soft tissue invasion, and distant metastasis. Due to old age or advanced local invasion, three patients did not undergo a complete thyroidectomy. After the mean follow-up period of 7.1 years, the nonremission rate, disease-specific mortality, and overall mortality did not differ significantly between patients that had undergone a total thyroidectomy and patients who had undergone less aggressive surgical treatments. Of the 626 patients, 335 patients had clinical stage 1 disease, without lymph node metastasis, soft tissue invasion, or distant metastasis. At the end of the follow-up period, there was no disease-specific mortality in these 335 patients; however, there were nine cases of nonthyroid cancer mortality. In addition, 17 of the 335 patients presented with recurrent disease after a thyroidectomy including 10 patients with lymph node metastasis and 2 patients with thyroid bed soft tissue recurrence.

After the mean follow-up period of 7.1 ± 5.3 years, 131 (20.9%) patients were diagnosed as having a nonremission status.  Table 3 shows the clinical characteristics of patients in remission and nonremission. The nonremission group was predominantly male and had larger tumor sizes, higher postoperative levels of serum Tg, a more advanced TNM stage, higher disease-specific and total mortality, and a lower percentage of patients in disease-free status. There were no statistically significant differences in age or surgical procedures between the remission and nonremission groups; however, patients in the nonremission group received higher accumulated doses of ^131^I and a higher percentage of patients underwent external radiotherapy.

**Figure 2:**
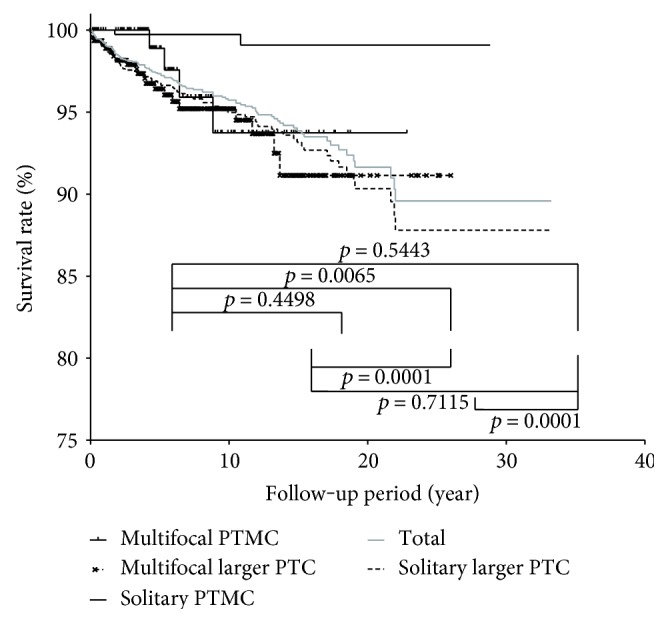
Kaplan-Meier survival curves of the four subject groups: multifocal PTMC, multifocal larger PTC, solitary PTMC, and solitary larger PTC. PTMC: papillary thyroid microcarcinoma.

Of the 626 patients with multifocal PTC, 25 (4%) patients died during a mean follow-up period of 7.1 ± 5.3 years. A comparison of risk factors between the cancer mortality and survival groups revealed that male sex, older age, larger tumor size, higher postoperative serum levels of Tg, and advanced TNM stage differed significantly between these groups (Table 4). Multivariant analyses were performed using the Cox proportional hazards regression model and revealed that only age differed significantly between the survival and mortality groups (Table 5). The 5-, 10-, and 20-year survival rates of the 2536 patients with PTC were 97.3%, 95.7%, and 91.6%, respectively.  Figure 2 shows the Kaplan-Meier survival curves of the patients in the multifocal PTMC, multifocal larger PTC, solitary PTMC, and solitary larger PTC groups. The 5-, 10-, and 20-year survival rates of the four groups were 98.9%, 96.4%, 99.7%, and 96.8%; 93.7%, 95.2%, 99.7%, and 95.0%; and 93.7%, 91.4%, 99.0%, and 90.3%, respectively. The survival rates of patients with solitary PTMC were significantly different from the survival rates of patients in the other three groups (Figure 2). There were no significant differences in survival rates between the other three groups.

## 4. Discussion

Multifocal PTC is the most frequently diagnosed multifocal, well-differentiated thyroid cancer, although follicular and medullary thyroid cancer may also present with PTC in the same patient [13]. Patients may be categorized as having multifocal PTMC, multifocal larger PTC, and mixed multifocal PTMC with larger tumors. In our study, one-quarter of the PTC cases were multifocal. Although the mean tumor size of the largest multifocal tumor was smaller than that in the solitary PTC group, the clinical and TNM stages were more advanced. In contrast to that in a recent study, the multifocal group in our study had a higher nonremission rate and a lower percentage of patients were disease-free when compared to the solitary PTC group [14]. These discordant results may due to the larger number of patients enrolled and the longer follow-up period in our study.

In our study, 23.5% of multifocal PTCs were microcarcinomas. This ratio was close to the reported proportion of PTMC in an earlier study [15]. The prognosis of multifocal PTC was better than the prognosis for multifocal follicular or Hurthle cell histology [16]. However, more data are required regarding the long-term therapeutic outcomes of multifocal PTC in microcarcinoma or larger tumors, as well as how unilateral or bilateral tumors and the number of tumors in multifocal carcinoma may affect treatment outcomes [1, 17]. Our study response to the consensus report of the European Society of Endocrine Surgeons suggests that prognosis might be impaired in clinical multifocal PTC, but the effect might be less or none in tumors < 1 cm [18]. Our results showed that multifocal papillary microcarcinoma had a lower relapse rate than larger tumors; however, there were no statistically significant differences in cancer and overall mortality after a mean follow-up of 7.1 ± 5.3 years.

Diagnosis of multifocal PTC may occur preoperatively by ultrasound and fine-needle aspiration cytology (FNAC), during surgery by a frozen section, and postoperatively by final histopathology examination. There are few reports concerning the preoperative diagnosis of multifocal PTC, especially PTMC. The main explanation is that FNAC is not recommended for thyroid nodules of less than 1 cm. However, preoperative diagnosis of multifocal PTC including the location and number of tumors may be important information for surgical strategy and decision-making [16]. A recent study showed that bilateral multifocal PTC had a worse prognosis than unilateral multifocal PTC [5]. Lymph node dissection may be indicated in cases of multifocal PTC [19]. In our study, analysis of patients that underwent a less than total thyroidectomy for multifocal PTC did not show a worse prognosis than that of patients who had undergone a total thyroidectomy.

In our study, the multifocal PTC group had a higher percentage of patients in nonremission status compared to the solitary PTC group. As reported previously, PTMC had a low percentage of patients in nonremission compared to that of patients with multifocal larger tumor PTC [2]. However, we also found that disease-specific mortality did not differ significantly between multifocal PTMC and larger tumor PTC groups. In contrast, patients with multifocal PTMC had a worse prognosis and disease-specific survival rates compared to patients with solitary PTMC. A higher percentage of soft tissue invasion and distant metastasis in multifocal PTMC are part of reasons with poor prognosis than that in solitary PTMC. The use of ^131^I ablation for the treatment of multifocal PTMC is questioned by the European consensus and American Thyroid Association (ATA) guidelines [18, 20]. In our study, 3.4% of multifocal PTMC had distant metastasis. A more aggressive postoperative remnant ablation and longer follow-up period than that of solitary PTMC are indicated.

Several studies have assessed the clonal origin of multifocal PTC [21–24]. However, the results were inconsistent. BRAF gene mutation combined with X chromosome inactivation analyses has been used to evaluate the clonal origins of tumors and has showed that bilateral, recurrent, and metastatic PTCs often arise from a single clone and that intrathyroid metastasis may play an important role in the development of bilateral tumors [21]. In contrast, assessment of multifocal thyroid tumors using genetic alteration analyses and miRNA profiling found that multifocal PTC did not necessarily evolve from single PTC progenitor foci [20]. In the clinic, different histological patterns like follicular variant and classical PTC in different thyroid lobes are not unusual. Both intrathyroid lymphatic spreading and different clonal origins may be present in multifocal PTC [24]. This study has several strengths, including a nearly 10-year follow-up period of a large number of patients, which has strengthened the conclusions. The patients enrolled were diagnosed and treated at a single medical center, which may make the data more consistent. However, due to the enrollment period of over 30 years, examination and therapeutic modalities may likely have changed over time.

## 5. Conclusion

Multifocal PTMC had a lower recurrence rate than multifocal larger tumor PTC; however, there was no difference in cancer-specific mortality rates. These patients need a follow-up because there is a risk of recurrence.

## Supplementary Material

Supplemental Table A. Clinical features of multifocal and solitary larger papillary thyroid carcinoma. Supplemental Table B. Multivariate analysis by Cox proportional hazards regression model for survival and mortality of all 2,536 PTC patients. Supplemental Table C. Clinical features of multifocal papillary thyroid cancer undergoing total thyroidectomy or less than total thyroidectomy surgery. Supplemental Table D. Clinical features of multifocal or solitary papillary thyroid microcarcinoma. Supplemental Table E. Multivariate analysis by Cox proportional hazards regression model for survival and mortality of all 2,536 patients with papillary thyroid carcinoma. Supplemental Figure A

## Figures and Tables

**Table 1:** Clinical features of multifocal or solitary papillary thyroid cancer.

Clinical characteristics	All patients	Multifocal	Solitary	*p* value
Patient number	2536 (100.0)	626 (24.7)	1910 (75.3)	
Gender, female	1996 (78.7)	499 (79.7)	1497 (78.4)	0.479
Age at diagnosis (year)	43.7 ± 14.0	46.5 ± 13.5	42.7 ± 14.0	<0.001
Age ≥ 45 yrs.	1174 (46.3)	355 (56.7)	819 (42.9)	<0.001
Mean tumor size (cm)	2.3 ± 1.6	2.1 ± 1.4	2.3 ± 1.6	0.003
1-month postoperative serum Tg level (ng/mL)	157.4 ± 1851.2	184.2 ± 1551.1	148.6 ± 1939.7	0.683
Variants				
Hashimoto (lymphocytic) thyroiditis	133 (5.2)	29 (4.6)	104 (5.4)	0.429
Follicular variant of PTC	382 (15.1)	99 (15.8)	293 (15.3)	0.776
Poorly differentiated + tall cell	36 (1.4)	8 (1.3)	28 (1.5)	0.730
Operative method				
Total thyroidectomy	2191 (86.4)	595 (95.0)	1596 (83.6)	<0.001
TNM stage				
Stage I	1772 (69.9)	390 (62.3)	1382 (72.4)	<0.001
Stages II–IV	764 (30.1)	236 (37.7)	528 (27.6)	
Nonremission	381 (15.0)	131 (20.9)	250 (13.1)	<0.001
Follow-up period (year)	9.4 ± 6.8	7.1 ± 5.3	10.2 ± 7.0	<0.001
Postoperative ^131^I accumulative dose (mCi)	130.6 ± 199.9	153.7 ± 222.2	123.0 ± 191.4	0.001
Radiation therapy	114 (4.5)	28 (4.5)	86 (4.5)	0.975
2nd primary cancer	180 (7.1)	49 (7.8)	131 (6.9)	0.413
Diabetes mellitus	210 (8.3)	61 (9.7)	149 (7.8)	0.126
Overall mortality	227 (9.0)	49 (7.8)	178 (9.3)	0.256
Cancer mortality	109 (4.3)	25 (4.0)	84 (4.4)	0.665
Disease-free	982 (38.7)	221 (35.3)	761 (39.8)	0.043
Lymph node metastasis	581 (22.9)	197 (31.5)	384 (20.1)	<0.001
Soft tissue invasion	520 (20.5)	153 (24.4)	367 (19.2)	0.005
Distant metastasis	112 (4.4)	39 (6.2)	73 (3.8)	0.011

Number (%); mean ± SD.

**Table 2:** Clinical features of multifocal papillary thyroid cancer in different tumor sizes.

Clinical characteristics	All patients	Tumor size ≤ 1.0 cm	Tumor size > 1.0 cm	*p* value
Patient number	626 (100.0)	147 (23.5)	479 (76.5)	
Gender, female	499 (79.7)	116 (78.9)	383 (80.0)	0.783
Age at diagnosis (year)	46.5 ± 13.5	47.1 ± 11.4	46.3 ± 14.1	0.541
Age ≥ 45 yrs.	355 (56.7)	90 (61.2)	265 (55.3)	0.207
1-month postoperative serum Tg level (ng/mL)	184.2 ± 1551.1	296.5 ± 2725.5	150.3 ± 941.6	0.331
Operative method				
Total thyroidectomy	595 (95.0)	130 (88.4)	465 (97.1)	<0.001
TNM stage				
Stage I	390 (62.3)	122 (83.0)	268 (55.9)	<0.001
Stages II–IV	236 (37.7)	25 (17.0)	211 (44.1)	
Nonremission	131 (20.9)	18 (12.2)	113 (23.6)	0.003
Residual	83 (13.3)	13 (8.8)	70 (14.6)	0.071
Relapsed	48 (7.7)	5 (3.4)	43 (9.0)	0.026
Follow-up period (year)	7.1 ± 5.3	6.6 ± 5.1	7.2 ± 5.4	0.245
Postoperative ^131^I accumulative dose (mCi) [range]	153.7 ± 222.2 [0.0–2050.0]	114.3 ± 176.7 [0.0–1350.0]	165.9 ± 233.0 [0.0–2050.0]	0.014
^131^I ≥ 30 mCi	569 (90.9)	127 (86.4)	442 (92.3)	0.030
^131^I < 30 mCi	57 (9.1)	20 (13.6)	37 (7.7)	
Radiation therapy	28 (4.5)	5 (3.4)	23 (4.8)	0.472
2nd primary cancer	49 (7.8)	11 (7.5)	38 (7.9)	0.859
Diabetes mellitus	61 (9.7)	12 (8.2)	49 (10.2)	0.460
Overall mortality	49 (7.8)	8 (5.4)	41 (8.6)	0.218
Cancer mortality	25 (4.0)	4 (2.7)	21 (4.4)	0.368
Disease-free	221 (35.3)	58 (39.5)	163 (34.0)	0.228
Lymph node metastasis	197 (31.5)	33 (22.4)	164 (34.2)	0.007
Soft tissue invasion	153 (24.4)	14 (9.5)	139 (29.0)	<0.001
Distant metastasis	39 (6.2)	5 (3.4)	34 (7.1)	0.105

Number (%); mean ± SD.

**Table 3:** Clinical features of multifocal papillary thyroid cancer in nonremission or remission.

Clinical characteristics	All patients	Nonremission	Remission	*p* value
Patient number	626 (100.0)	131 (20.9)	495 (79.1)	
Gender, female	499 (79.7)	84 (64.1)	415 (83.8)	<0.001
Age at diagnosis (year)	46.5 ± 13.5	47.8 ± 17.1	46.1 ± 12.4	0.214
Mean tumor size (cm)	2.1 ± 1.4	2.6 ± 1.7	2.0 ± 1.3	<0.001
1-month postoperative serum Tg level (ng/mL)	184.2 ± 1551.1	770.2 ± 3286.7	25.3 ± 102.8	<0.001
Operative method				
Total thyroidectomy	595 (95.0)	127 (96.9)	468 (94.5)	0.260
Less than total thyroidectomy	31 (5.0)	4 (3.1)	27 (5.5)	
TNM stage				
Stage I	390 (62.3)	53 (40.5)	337 (68.1)	<0.001
Stage II	58 (9.3)	15 (11.5)	43 (8.7)	0.332
Stage III	67 (10.7)	14 (10.7)	53 (10.7)	0.995
Stage IV	111 (17.7)	49 (37.4)	62 (12.5)	<0.001
Follow-up period (year)	7.1 ± 5.3	7.4 ± 5.5	7.0 ± 5.3	0.478
Postoperative ^131^I accumulative dose (mCi)	153.7 ± 222.2	384.2 ± 366.4	92.8 ± 95.5	<0.001
Radiation therapy	28 (4.5)	27 (20.6)	1 (0.2)	<0.001
2nd primary cancer	49 (7.8)	15 (11.5)	34 (6.9)	0.083
Diabetes mellitus	61 (9.7)	13 (9.9)	48 (9.7)	0.938
Overall mortality	49 (7.8)	29 (22.1)	20 (4.0)	<0.001
Cancer mortality	25 (4.0)	24 (18.3)	1 (0.2)	<0.001
Disease-free	221 (35.3)	16 (12.2)	205 (41.4)	<0.001

Number (%); mean ± SD.

**Table 4:** Clinical features of multifocal papillary thyroid cancer in cancer mortality.

Clinical characteristics	All patients	Cancer mortality	Survival	*p* value
Patient number	626 (100.0)	25 (4.0)	601 (96.0)	
Gender, female	499 (79.7)	9 (36.0)	490 (81.5)	<0.001
Age at diagnosis (year)	46.5 ± 13.5	61.0 ± 13.8	45.9 ± 13.2	<0.001
Mean tumor size (cm)	2.1 ± 1.4	3.2 ± 2.3	2.0 ± 1.3	<0.001
1-month postoperative serum Tg level (ng/mL)	184.2 ± 1551.1	2060.6 ± 6608.4	112.8 ± 834.4	<0.001
Operative method				
Total thyroidectomy	595 (95.0)	25 (100.0)	570 (94.8)	0.244
Less than total thyroidectomy	31 (5.0)	—	31 (5.2)	
TNM stage				
Stage I	390 (62.3)	2 (8.0)	388 (64.6)	<0.001
Stages II–IV	236 (37.7)	23 (92.0)	213 (35.4)	
Nonremission	131 (20.9)	24 (96.0)	107 (17.8)	<0.001
Follow-up period (year)	7.1 ± 5.3	4.9 ± 3.9	7.2 ± 5.4	0.032
Postoperative ^131^I accumulative dose (mCi)	153.7 ± 222.2	341.1 ± 317.4	146.0 ± 213.8	<0.001
Radiation therapy	28 (4.5)	14 (56.0)	14 (2.3)	<0.001
2nd primary cancer	49 (7.8)	2 (8.0)	47 (7.8)	0.974
Diabetes mellitus	61 (9.7)	—	61 (10.1)	0.094
Lymph node metastasis	197 (31.5)	14 (56.0)	183 (30.4)	0.007
Soft tissue invasion	153 (24.4)	18 (72.0)	135 (22.5)	<0.001
Distant metastasis	39 (6.2)	14 (56.0)	25 (4.2)	<0.001

Number (%); mean ± SD.

**Table 5:** Multivariate analysis by the Cox proportional hazards regression model for survival and mortality.

	*β* coefficient	Hazard ratio	95% confidence interval		*p* value
			Lower bound	Upper bound	
Age (year)	0.013	1.013	1.006	1.020	0.0003
Sex (female versus male)	0.174	1.190	0.956	1.481	0.1190
Tumor size (cm)	−0.062	0.940	0.882	1.001	0.0534
Postoperative serum Tg level after 1 month (ng/mL)	0.000	1.000	1.000	1.000	0.5640
Metastases (soft tissue invasion/distant metastases)	−0.113	0.893	0.784	1.016	0.0863
