# Supplementary material for: Therapeutic Outcomes of Patients with Multifocal Papillary Thyroid Microcarcinomas and Larger Tumors
Source: Int J Endocrinol. 2017 May 31;2017:4208178. doi: 10.1155/2017/4208178 (PMC5470030; doi:10.1155/2017/4208178)
Supplement: Supplementary file 1 — Supplemental Table A. Clinical features of multifocal and solitary larger papillary thyroid carcinoma. Supplemental Table B. Multivariate analysis by Cox proportional hazards regression model for survival and mortality of all 2,536 PTC patients. Supplemental Table C. Clinical features of multifocal papillary thyroid cancer undergoing total thyroidectomy or less than total thyroidectomy surgery. Supplemental Table D. Clinical features of multifocal or solitary papillary thyroid microcarcinoma. Supplemental Table E. Multivariate analysis by Cox proportional hazards regression model for survival and mortality of all 2,536 patients with papillary thyroid carcinoma. Supplemental Figure A [file 4208178.f1.pdf]

SUPPLEMENTAL TABLE A. **Clinical features of multifocal and solitary larger papillary thyroid carcinoma.**

**Clinical characteristic**	**Multifocal**	**Solitary**	***p* value**
Patient number	479	1,490	
Gender, Female	383 (80.0)	1,141 (76.6)	0.124
Age at diagnosis (year)	46.3 ± 14.1	42.2 ± 14.2	<0.001
Age≥ 45 yrs.	265 (55.3)	605 (40.6)	<0.001
1-month postoperative serum Tg level (ng/mL)	150.3 ± 941.6	171.8 ± 2143.2	0.835
Operative method			
Total thyroidectomy	465 (97.1)	1,359 (91.2)	<0.001
TNM stage			
Stage I	268 (55.9)	996 (66.8)	<0.001
Stage II	56 (11.7)	173 (11.6)	0.962
Stage III	55 (11.5)	111 (7.4)	0.006
Stage IV	100 (20.9)	210 (14.1)	<0.001
Non-remission	113 (23.6)	232 (15.6)	<0.001
Residual	70 (14.6)	130 (8.7)	<0.001
Relapsed	43 (9.0)	102 (6.8)	0.120
Follow-up period (year)	7.2 ± 5.4	10.6 ± 7.2	<0.001
Post-operative ^{131}I accumulative dose (mCi) [range]	165.9 ± 233.0 [0.0-2050.0]	139.2 ± 196.3 [0.0-2787.1]	0.014
^{131}I≥ 30 mCi	442 (92.3)	1383 (92.8)	0.691
^{131}I< 30 mCi	37 (7.7)	107 (7.2)	
Radiation therapy	23 (4.8)	79 (5.3)	0.667
2nd primary cancer	38 (7.9)	101 (6.8)	0.391
Diabetes mellitus	49 (10.2)	106 (7.1)	0.028
Overall mortality	41 (8.6)	156 (10.5)	0.225
Cancer mortality	21 (4.4)	82 (5.5)	0.339
Disease free	163 (34.0)	642 (43.1)	<0.001
Lymph node metastasis	164 (34.2)	344 (23.1)	<0.001
Soft tissue invasion	139 (29.0)	351 (23.6)	0.016
Distant metastasis	34 (7.1)	66 (4.4)	0.021

Number (%); Mean ± SD

SUPPLEMENTAL TABLE B. Multivariate analysis by Cox proportional hazards regression model for survival and mortality of all 2,536 PTC patients.

	β coefficient	Hazard ratio	95% confidence interval		*p* value
			Lower bound	Upper bound	
Age (year)	.015	1.015	1.012	1.019	0.0000
Sex (Female vs. Male)	.123	1.131	1.020	1.255	0.0200
Tumor size (cm)	-.098	.906	.879	.935	0.0000
Post-operative serum Tg level after 1 month (ng/mL)	.000	1.000	1.000	1.000	0.4423
Metastases (lymph node/soft tissue invasion/distant metastases)	-.019	.981	.935	1.029	0.4235

SUPPLEMENTAL TABLE C. Clinical features of multifocal papillary thyroid cancer undergoing total thyroidectomy or less than total thyroidectomy surgery.

Clinical characteristic	All patients	Total thyroidectomy	Less than total thyroidectomy	*p* value
Patient number	626 (100.0)	595 (95.0)	31 (5.0)	
Gender, Female	499 (79.7)	472 (79.3)	27 (87.1)	0.294
Age at diagnosis (year)	46.5 ± 13.5	46.6 ± 13.5	44.4 ± 14.7	0.379
Mean tumor size (cm)	2.1 ± 1.4	2.1 ± 1.4	1.1 ± 0.8	<0.001
1-month postoperative serum Tg level (ng/mL)	184.2 ± 1551.1	182.3 ± 1579.6	221.4 ± 804.5	0.895
TNM stage				
Stage I	390 (62.3)	363 (61.0)	27 (87.1)	0.003
Stage II	58 (9.3)	57 (9.6)	1 (3.2)	0.234
Stage III	67 (10.7)	66 (11.1)	1 (3.2)	0.167
Stage IV	111 (17.7)	109 (18.3)	2 (6.5)	0.092
Non-remission	131 (20.9)	127 (21.3)	4 (12.9)	0.260
Follow-up period (year)	7.1 ± 5.3	7.1 ± 5.4	7.3 ± 4.3	0.866
Post-operative ^{131}I accumulative dose (mCi)	153.7 ± 222.2	157.4 ± 225.0	82.9 ± 140.4	0.069
Radiation therapy	28 (4.5)	28 (4.7)	-	0.217
2nd primary cancer	49 (7.8)	45 (7.6)	4 (12.9)	0.281
Diabetes mellitus	61 (9.7)	59 (9.9)	2 (6.5)	0.526
Overall mortality	49 (7.8)	48 (8.1)	1 (3.2)	0.328
Cancer mortality	25 (4.0)	25 (4.2)	-	0.244
Disease free	221 (35.3)	214 (36.0)	7 (22.6)	0.128
Lymph node metastasis	197 (31.5)	196 (32.9)	1 (3.2)	0.001
Soft tissue invasion	153 (24.4)	152 (25.5)	1 (3.2)	0.005
Distant metastasis	39 (6.2)	38 (6.4)	1 (3.2)	0.478

Number (%); Mean ± SD

SUPPLEMENTAL TABLE D. Clinical features of multifocal or solitary papillary thyroid microcarcinoma.

Clinical characteristic	Multifocal	Solitary	*p* value
Patient number	147	420	
Gender, Female	116 (78.9)	356 (84.8)	0.102
Age at diagnosis (year)	47.1 ± 11.4	44.8 ± 12.8	0.055
Age≥ 45 yrs.	90 (61.2)	214 (51.0)	0.032
1-month postoperative serum Tg level (ng/mL)	296.5 ± 2725.5	62.2 ± 811.0	0.132
Operative method			
Total thyroidectomy	130 (88.4)	237 (56.4)	<0.001
TNM stage			
Stage I	122 (83.0)	386 (91.9)	0.002
Stage II	2 (1.4)	2 (0.5)	0.270
Stage III	12 (8.2)	17 (4.0)	0.051
Stage IV	11 (7.5)	15 (3.6)	0.051
Non-remission	18 (12.2)	18 (4.3)	0.001
Residual	13 (8.8)	9 (2.1)	<0.001
Relapsed	5 (3.4)	9 (2.1)	0.397
Follow-up period (year)	6.6 ± 5.1	8.9 ± 6.4	<0.001
Post-operative ^{131}I accumulative dose (mCi) [range]	114.3 ± 176.7	65.5 ± 160.2	0.002
^{131}I≥ 30 mCi	127 (86.4)	248 (59.0)	<0.001
^{131}I< 30 mCi	20 (13.6)	172 (41.0)	
Radiation therapy	5 (3.4)	7 (1.7)	0.209
2nd primary cancer	11 (7.5)	30 (7.1)	0.891
Diabetes mellitus	12 (8.2)	43 (10.2)	0.464
Overall mortality	8 (5.4)	22 (5.2)	0.924
Cancer mortality	4 (2.7)	2 (0.5)	0.022
Disease free	58 (39.5)	119 (28.3)	0.012
Lymph node metastasis	33 (22.4)	40 (9.5)	<0.001
Soft tissue invasion	14 (9.5)	16 (3.8)	0.008
Distant metastasis	5 (3.4)	7 (1.7)	0.209

Number (%); Mean ± SD

SUPPLEMENTAL TABLE E. Multivariate analysis by Cox proportional hazards regression model for survival and mortality of all 2,536 patients with papillary thyroid carcinoma .

	β coefficient	Hazard ratio	95% confidence interval		*p* value
			Lower bound	Upper bound	
Age (year)	0.014	1.014	1.011	1.017	0.0000
Sex (Female vs. Male)	0.141	1.151	1.038	1.277	0.0078
Tumor size (cm)	-0.090	0.914	0.886	0.942	0.0000
Post-operative serum Tg level after 1 month (ng/mL)	0.000	1.000	1.000	1.000	0.6728
Metastases (lymph node vs. soft tissue invasion vs. distant metastses)	-0.039	0.962	0.917	1.009	0.1076
Solitary vs. Multifocal PTC*	0.499	1.646	1.493	1.815	0.0000

PTC*: papillary thyroid carcinoma

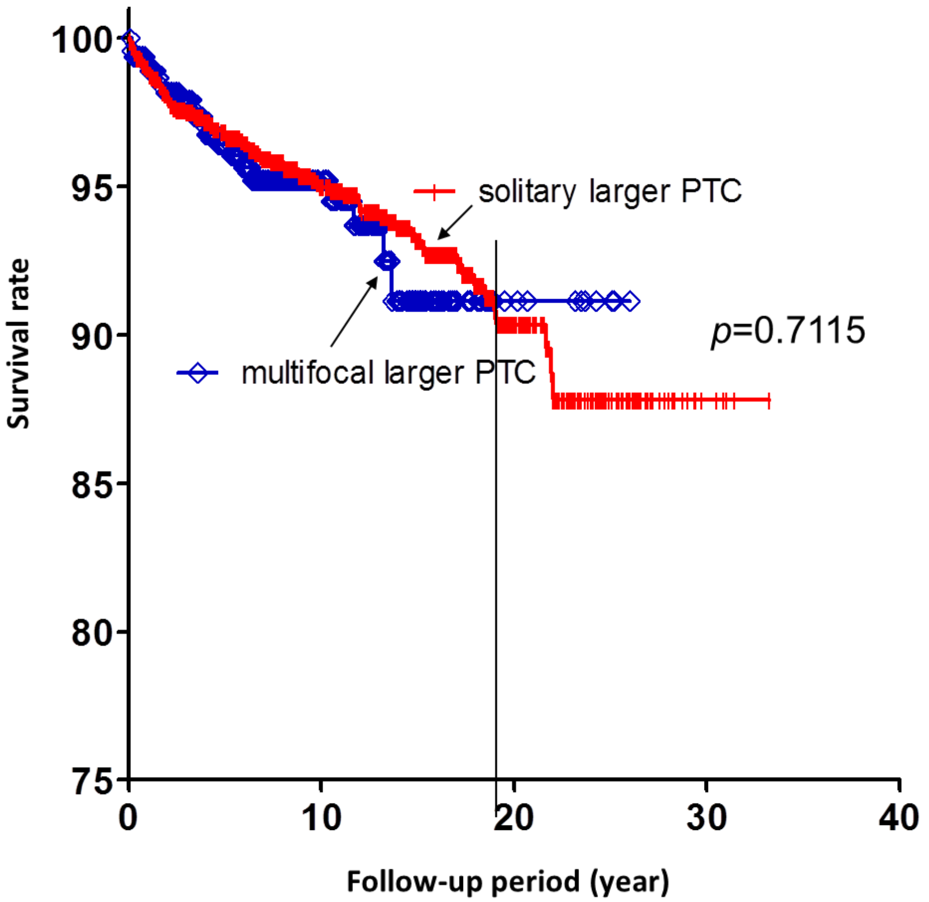

Supplemental Figure A
